# Highlights from the 3rd international HIV/viral hepatitis Co-infection meeting - HIV/viral hepatitis: improving diagnosis, antiviral therapy and access

**DOI:** 10.1186/s41124-017-0025-0

**Published:** 2017-04-20

**Authors:** Tongai G. Maponga, Rachel Matteau Matsha, Sébastien Morin, Andrew Scheibe, Tracy Swan, Isabelle Andrieux-Meyer, C. Wendy Spearman, Marina B. Klein, Jürgen Kurt Rockstroh

**Affiliations:** 1 Division of Medical Virology, University of Stellenbosch, Faculty of Medicine and Health Sciences, Stellenbosch, South Africa; 2 Urban Futures Centre, Durban University of Technology, Durban, South Africa; 3 HIV Programmes and Advocacy, International AIDS Society, Geneva, Switzerland; 4 TB/HIV Care Association and Desmond Tutu HIV Centre, Cape Town, South Africa; 5 Treatment Action Group, New York, USA; 6 Médecins Sans Frontières, Access Campaign, Geneva, Switzerland; 7 Division of Hepatology, Department of Medicine, University of Cape Town, Cape Town, South Africa; 8 Chronic Viral Illness Service, McGill University Health Centre, Montreal, Canada; 9 Department of Medicine I, University Hospital Bonn, Bonn, Germany

**Keywords:** HIV/viral hepatitis co-infection, Hepatitis B, Hepatitis C, Epidemiology, Prevention, Access and equity of treatment, 21st International AIDS Conference

## Abstract

The International AIDS Society convened the 3rd International HIV/Viral Hepatitis Co-Infection Meeting on 17 July 2016 as part of the pre-conference program preceding the 21st International AIDS Conference held in Durban, South Africa. The meeting brought together a diversity of scientific, technical and community interests to discuss opportunities and challenges for increased prevention, diagnosis and treatment of viral hepatitis in people living with HIV, particularly in low- and middle-income settings.

The objectives of the meeting were:

i. To review the latest therapeutic developments in viral hepatitis;
ii. To identify challenges such as high cost of medications for hepatitis C virus (HCV) and risk of developing viral resistance, and successes, such as the provision of HCV treatment in community-based settings, movements to reduce drug costs and increasing access, in relation to scaling up diagnosis, screening, antiviral treatment and prevention of viral hepatitis;
iii. To advance the agenda for elimination of viral hepatitis as a public health problem.

Discussions centred around the six key interventions outlined by the World Health Organization Global Health Sector Strategy on Viral Hepatitis 2016–2021: hepatitis B virus (HBV) vaccination (including birth dose); safe injection practices plus safe blood; harm reduction among people who inject drugs; safer sex practices; hepatitis B treatment; and hepatitis C cure.

This article summarizes the main issues and findings discussed during the pre-conference meeting. One of the recommendations from the meeting delegates is universal implementation of birth dose vaccination for HBV without further delay to prevent mother-to-child transmission of infection. There is also the need to implement screening and treatment of hepatitis among pregnant women. A call was made for concerted efforts to be put together by all stakeholders towards addressing some of the structural barriers, including criminalization of drug use, discrimination and stigma that people living with viral hepatitis face. Finally, the need for greater advocacy was highlighted to enable access to therapy of viral hepatitis at lower cost than currently prevails. Implementation of these resolutions will help in achieving the target of eliminating viral hepatitis as a public health threat.

## Introduction

The 3rd International HIV/Viral Hepatitis Co-Infection Meeting (http://www.iasociety.org/co-infections/hepatitis), chaired by Wendy Spearman, Marina Klein (in absentia) and Jürgen Rockstroh, convened some 400 participants from a variety of backgrounds, including researchers, epidemiologists, clinicians, funders, biomedical industry representatives, policy makers, health activists and people living with viral hepatitis and/or HIV.

The meeting was organized around six major themes:

i. Hepatitis B virus (HBV) topics
ii. Challenges in HBV management in resource-limited settings
iii. Hepatitis C virus (HCV) epidemiology: The knowns and the unknowns
iv. HCV treatment update: A moving target
v. New tools, new technologies
vi. Panel discussion - Accessing antivirals: Overcoming remaining challenges.

Speakers delivered oral presentations, and selected authors presented posters on issues that included: HBV epidemiology and outcomes; HCV epidemiology and natural history; HCV testing, monitoring and management, and HCV treatment access. This report presents the key points of the meeting and concludes with the main outcomes and the recommendations made by delegates.

## Background

Global estimates suggest that 240 million people were living with chronic hepatitis B as of 2005 [1], while 80 million (95% confidence interval: 64–103) have chronic HCV viraemia [2]. Despite the high numbers of infected people, less than 5% of people living with chronic viral hepatitis are aware that they are infected because of the insidious nature of viral hepatitis infections and lack of access to affordable diagnostics [3]. Furthermore, according to statistics from the 2013 Global Burden of Disease Study, an estimated 1.45 million (95% uncertainty interval: 1.38–1.54) people die each year from viral hepatitis-related diseases, with 96% of these being due to HBV and HCV infections [4]. The number of people who die from viral hepatitis is greater than those who die from HIV, tuberculosis or malaria; yet viral hepatitis (also including hepatitis A, D and E) does not get as much attention [4]. Six to 10 million people are newly infected with viral hepatitis annually, and this is despite the existence of effective preventative measures and therapeutic treatments, such as HBV vaccine and antivirals, and direct acting antivirals (DAAs) for cure of HCV [3].

There is an urgent need to strengthen equitable access to prevention, screening, diagnosis and treatment services to the most affected regions, particularly in low- and middle-income settings, such as sub-Saharan Africa and Central and East Asia. In May 2016, the World Health Assembly adopted the Global Health Sector Strategy on Viral Hepatitis 2016–2021, whose objectives are aligned with those of the Sustainable Development Goals. The strategy’s long-term vision is to eliminate viral hepatitis as a public health threat by 2030 through the reduction of new viral hepatitis infections by 90% and the reduction of deaths related to viral hepatitis by 65% from the 2015 figures [3].

It is in this context that the 3rd International HIV/Viral Hepatitis Co-Infection Meeting opened with coverage of the epidemiology and burden of disease related to HIV, HBV and HCV infections. Despite viral hepatitis being one of the 10 leading causes of mortality and morbidity worldwide, there is an acute lack of global awareness about the severity of the problem and a lack of commitment to combat and eliminate the disease.

The burden and consequences of viral hepatitis are not evenly distributed worldwide: Oceania, sub-Saharan Africa and Asia have the highest viral hepatitis-related mortality rates [4]. Moreover, it is well recognized that co-infection with HIV and viral hepatitis is associated with worse outcomes than being infected with HBV or HCV alone, particularly with advanced immunodeficiency [5, 6]. Patients co-infected with HIV and viral hepatitis show rapid progression to cirrhosis and early presentation with hepatocellular carcinoma (HCC) compared with those infected with viral hepatitis alone [7, 8]. Improved availability and access to appropriate diagnosis and treatment is needed to reduce the number of people with viral hepatitis-related liver diseases. The elimination of viral hepatitis will require strong partnerships between affected communities, professional and community organizations, national departments of health, researchers, health care providers and the biomedical industry.

### Hepatitis B topics: epidemiology, prevention and treatment

HIV/HBV co-infection remains a global public health challenge. In HBV endemic countries, childhood-acquired hepatitis B infections usually precede HIV acquisition in adulthood. Sub-Saharan Africa faces the dual challenge of having both the highest prevalence of HIV infection and also high endemicity of hepatitis B [1, 9]. The dual burden of HBV and HIV infection poses a challenge in that mortality from liver disease due to HIV/HBV co-infection has been shown to be higher than due to HIV/HCV co-infection, as shown by data from the Multicenter AIDS Cohort Study where the liver-related mortality among HIV-infected men with chronic hepatitis B was 13.4 per 1000 person-years compared with 7.2 per 1000 person-years in those with hepatitis C [10].

HBV seroprevalence and transmission risks vary geographically. In low prevalence countries, hepatitis B is usually acquired in adulthood either sexually or parenterally, e.g., from needle stick-injuries. In contrast, in HBV endemic countries, mother-to-child transmission (MTCT) and early childhood infection from infected older siblings and playmates are the main routes of acquisition and are responsible for the chronicity of infection. Up to 90% of neonates born to HBV e antigen positive or highly viraemic mothers (with HBV DNA >200,000 IU/ml) and 20–50% with childhood infection (<5 years of age) will develop chronic hepatitis B compared to <5% of those who acquire hepatitis B as adults (>20 years of age). Thus, in HBV endemic countries, interrupting early transmission is key to breaking the cycle of ongoing HBV infection.

Prevention of MTCT of HBV and early childhood acquisition significantly reduces the number of new infections and eventually leads to the elimination of hepatitis B amongst neonates who serve as the reservoir of infections and have a propensity for establishing chronicity. This can be achieved through a combination of third trimester antiviral prophylaxis, hepatitis B hyperimmune globulin (HBIG), hepatitis B birth dose (HepB-BD) vaccination and ensuring full HBV vaccine coverage as well as safe delivery practices.

The combined administration of HBIG and HepB-BD monovalent vaccine within 24 h of delivery prevents HBV MTCT in 80–95% cases [11, 12]. In resource-rich settings, giving HBIG to infants born to pregnant women with high HBV viral loads, in addition to the HepB-BD vaccine, is standard of care to prevent HBV MTCT. However, HBIG is expensive and is not readily available in most HBV endemic countries and thus initiation of prophylactic nucleoside analogue antiviral therapy such as tenofovir in the third trimester should be considered to further reduce the risk of MTCT. However, emphasis must be placed on the administration of the HepB-BD monovalent vaccine within 24 h of delivery followed by the full HBV vaccine schedule (either as two or three additional monovalent vaccines or as a multivalent vaccine given according to the routine Expanded Programme of Immunisation schedule). However, in 2014, only 96 of 194 WHO countries (49%) reported offering HepB-BD vaccine as part of their national immunization programmes and <38% of babies born worldwide received the HepB-BD vaccine within 24 h after birth; and the WHO/UNICEF 2015 report estimated that only 80% infants received full vaccine coverage [13, 14].

The impact of the rollout of the multivalent vaccines has hampered the availability of the HBV monovalent vaccine that is needed if countries are to implement the WHO’s 2009 recommendation for a HepB-BD vaccine. The fact that multivalent vaccines improve timeliness and coverage rate of vaccination is not in doubt as these are more acceptable because of the reduced number of injections [15–17]. However, these improvements have not entirely helped eliminate perinatal transmission of hepatitis B in at-risk children who are not getting the much-needed birth dose. Perhaps other countries could learn from countries such as Colombia, where a HBV monovalent birth dose is administered in addition to the three doses of the multivalent vaccine [18]. There is little to non-existent evaluation of a serological response following most vaccines in children. However, post-vaccine serological testing (hepatitis B surface antigen, HBsAg, and anti-HBs antibody) of HBV-exposed infants at 9–12 months (or 1 or 2 months after their last HBV vaccine, if the vaccine series is delayed) as recommended in the United States by the Center for Disease Control and Prevention (CDC), enables confirmation of a serological response (anti-HBs levels >10 mIU/mL) to the vaccine and identification of infected infants [19]. Infants who are HBsAg negative with anti-HBs levels <10 mIU/mL require repeat vaccination and those who have become HBsAg positive need to be linked to care. However, there are concerns regarding feasibility and cost of post-vaccine serological testing in resource-limited settings.

All women who are pregnant should undergo antenatal screening for HBsAg, but this is currently not being done in many countries. This should be performed at the same time as screening for HIV. Screening for HBsAg could be achieved by rapid, point-of-care testing as a way of identifying women who are infected with HBV and need to receive appropriate intervention to prevent MTCT of HBV as well as linkage to ongoing care post-delivery. Identification of HBsAg positive pregnant women provides further opportunities to screen, vaccinate and identify potentially infected partners, siblings and children thereby identifying clusters of HBV infection and breaking cycles of HBV infection within families. Unfortunately, there are currently limited options for paediatric treatment of hepatitis B. Paediatric clinical trials are necessary to evaluate newer drugs, such as tenofovir alafenamide, that are less nephrotoxic and do not have as adverse an impact on bone mineralization.

The inclusion of HBV screening into the routine antenatal testing schedule in highly endemic areas is important, but this requires the prioritization of HBV mono-infection, as there is a tendency to only be concerned about hepatitis B in the context of HIV. This is exemplified by the fact that the increasing availability of fixed-dose combinations (tenofovir, lamivudine/emtricitabine and efavirenz) for HIV therapy has become a limiting factor for access to anti-HBV therapeutic options, such as tenofovir that are needed by HBV-mono-infected patients. As a result, governments in resource-limited settings have to pay more in order to have the same drugs that are subsidized for HIV when treating hepatitis B. It appears that for the HBV-mono-infected patients, being HIV negative is ironically “a disadvantage” as it excludes many patients from accessing effective treatment at a reasonable cost [20]. For example, the current situation leads to only HIV/HBV-co-infected women benefitting from HIV treatment programs because of dually active antiretroviral therapy regimens that are effective against HIV and HBV, while HBV-mono-infected pregnant women are neglected and left at risk of transmitting infection to their babies. Routine screening and vaccination of at-risk individuals is also important as is the treatment of HBV infected individuals with active disease, but many HBV-mono-infected individuals fail to access antiviral therapy (tenofovir). Although the tools to effectively implement elimination strategies exist, they remain unequally distributed and are not easily accessible where they are needed most, particularly in resource-limited settings.

Despite progress made in terms of development of treatment and vaccination programs for viral hepatitis, the emergence of antiviral resistance mutations poses a threat, especially from patients with high viral loads. These mutations could be missed by diagnostic tests, cause reactivation of previous controlled HBV infections and could also result in vaccine escape, thereby permitting infection [21]. The transmission of resistant HBV viruses has been reported in several cases and could have an impact on the subsequent efficacy of HBV therapeutic regimens [22, 23]*.* Fortunately, the public health impact of these immune escape mutations appears to be limited for now, but more research is needed to improve the detection of drug-induced resistance and the related treatment failure. There is also a need for surveillance efforts to detect the emergence of these mutations.

There is also a need to update the current understanding of the natural history of HBV and the associated disease phases. The current understanding, especially of the *immune tolerant* phase, seems outdated and negatively impacts on patient care [24, 25]. This is because data shows that some perinatally infected children exhibit significant liver disease despite being classified as being in the *immune tolerant* phase, which is normally associated with minimal liver disease [26, 27]. There is also evidence of clonal repopulation of hepatocytes in the *immune toleran*t phase in some patients, suggestive of ongoing immune activity that results in killing of infected hepatocytes. There is thus a need for strengthened data collection in high-prevalence areas to improve our understanding of the natural history of hepatitis B.

In terms of HBV-related liver diseases, there is a need for routine surveillance for HCC and other liver-related complications of chronic hepatitis B infection. HCC was the second most common cause of cancer mortality worldwide, according to the 2012 GLOBOCAN data, but its management is currently poor [28]. There is an increase in the number of deaths linked to liver diseases, including HCC, and a noted increased incidence of HCC in people living with HIV who have HBV and/or HCV co-infection [8, 29]. Therefore, there is a need to refine the surveillance intervals in individuals with HIV co-infection because HCC in these patients tends to be more aggressive and grows more rapidly compared with those with HBV mono-infection. Current HCC surveillance recommendations are that there should be 6–12-month screening intervals using ultrasound [30, 31]. For example, the European Association for Study of the Liver (EASL) Guidelines for HCC screening in HIV and HCV/HBV co-infected individuals are similar to HCV and HBV mono-infected patients with established cirrhosis i.e., 6 monthly ultrasounds and alpha-fetoprotein levels [32]. In Africa, where access to ultrasound is limited, there may still be a role for alpha-fetoprotein in HCC surveillance. As the risk of HCC is increased in HIV and HBV/HCV co-infected individuals, is more aggressive and occurs at a younger age, there has been some discussion about shortening the length of screening intervals [33]. However, this may prove to be a challenge, especially when the current surveillance recommendations for HCC are not standardized nor systematically implemented in many countries. On a health system level, there is a challenge in the short to medium term to define delivery strategies for these screening and surveillance programs.

Finally, there are unmet challenges in hepatitis B therapy, such as the development of a cure. The discovery of the attachment receptor for HBV and the RNA-guided clustered regulatory interspaced short palindromic repeats (CRISPR) and CRISPR associated (Cas) protein endonucleases has led to the development of therapeutics aimed at achieving cure of infected patients [34]. Some therapeutic efforts for hepatitis B cure are geared towards elimination of HBV’s covalent closed circular DNA while others are focused on achieving global immune restoration [35].

### Hepatitis C topics: epidemiology, diagnostics and treatment

An estimated 110 million people have antibodies to HCV, which are considered serological evidence of current or past infection with hepatitis C [2]. Central and East Asia regions have high prevalence of HCV of above 3.5% in the general population, while sub-Saharan Africa is considered to have a moderate prevalence of between 1.5 and 3.5% [2].

People who inject drugs (PWID) are frequently marginalized by society and where coverage of needle and syringe programmes and opioid substitution therapy are limited, are at high risk of acquiring HIV and HCV infection [36]. PWID, particularly in resource-limited settings, are often unable to access testing and treatment services where they exist because of structural barriers, including the criminalization of drug use (i.e., policing), discrimination and stigma [37, 38]. While there are gaps in HCV epidemiology in Africa and other affected regions, populations of PWID do exist and are often underestimated, especially in eastern sub-Saharan Africa [39]. Limited data regarding HCV and HIV infections (and co-infections) among PWID is an obstacle to improving access and provision of quality treatment. There are very few African countries with harm reduction programs that include needle and syringe programs and/or opioid substitution therapy. Where harm reduction services exist, the programs are implemented by non-governmental organizations, which might indicate the lack of prioritization of services for PWID by governments of resource-limited countries [40]. These programs could play a positive role in increasing access to viral hepatitis-related services and, at the same time, help in reducing new HIV infections [41].

Other challenges include the absence of comprehensive country-specific public health policies for prevention, diagnosis and treatment of viral hepatitis, the restricted registration and high prices of DAAs rendering these effective drugs unavailable and unaffordable where they are needed, and the expensive and centralized diagnostic platforms that cannot be used in remote settings. Decentralized diagnostic platforms using economical and quality-assured rapid tests (serologic and RNA based) are important in facilitating linkage to care and treatment. By increasing the turnaround time for results, fewer patients would be lost to follow up [42]. The effectiveness of decentralized testing has been shown in HIV testing and treatment programs around the world. More point-of-care and near point-of-care diagnostic tools are being developed and becoming available, HCV programmes should ensure prompt and at-scale implementation, as well as effective linkage to care.

Given the availability of newly developed curative therapies, the elimination of HCV has become a real and achievable goal. This will, however, necessitate a strategic multifaceted approach. More specifically, the following will be required: increased testing; high-quality harm reduction services; improved quality of formal and informal health services; increased access to affordable treatment; development of a vaccine; and combatting stigma and discrimination against people living with hepatitis C. The role of resistance and resistance testing for managing hepatitis C in light of the availability of DAAs for treating hepatitis C must be carefully considered when feasible. Failure to achieve a sustained virological response using interferon-free DAAs usually involves HCV variants resistant to one or more DAAs. However, the addition of ribavirin or extending treatment duration increases the frequency with which a sustained virologic response is achieved [43]. Most of the anti-hepatitis C drug research and development has been targeted against HCV genotype 1 because the highest numbers of HCV genotype 1 infection are found in high-income countries [2, 44]. Unmet needs remain for genotype 3 in particular. Pangenotypic therapies would facilitate treatment, particularly in resource-limited settings, as these could be used without costly pre-treatment genotype assessment.

Although scaling up of treatment of hepatitis C is a positive step, this alone is insufficient as awareness of the infection is insufficient, especially among populations at high risk, such as PWID [45]. Treatment programs must ensure that there is enough education of patients, in addition to access to opioid substitution therapy and harm reduction programs and services, to avoid re-infections. In order to increase knowledge of hepatitis C, we need to develop and implement high-profile awareness and advocacy campaigns on viral hepatitis, as well as prevention campaigns and harm reduction programs, as has been done for HIV.

**Fig. 1:**
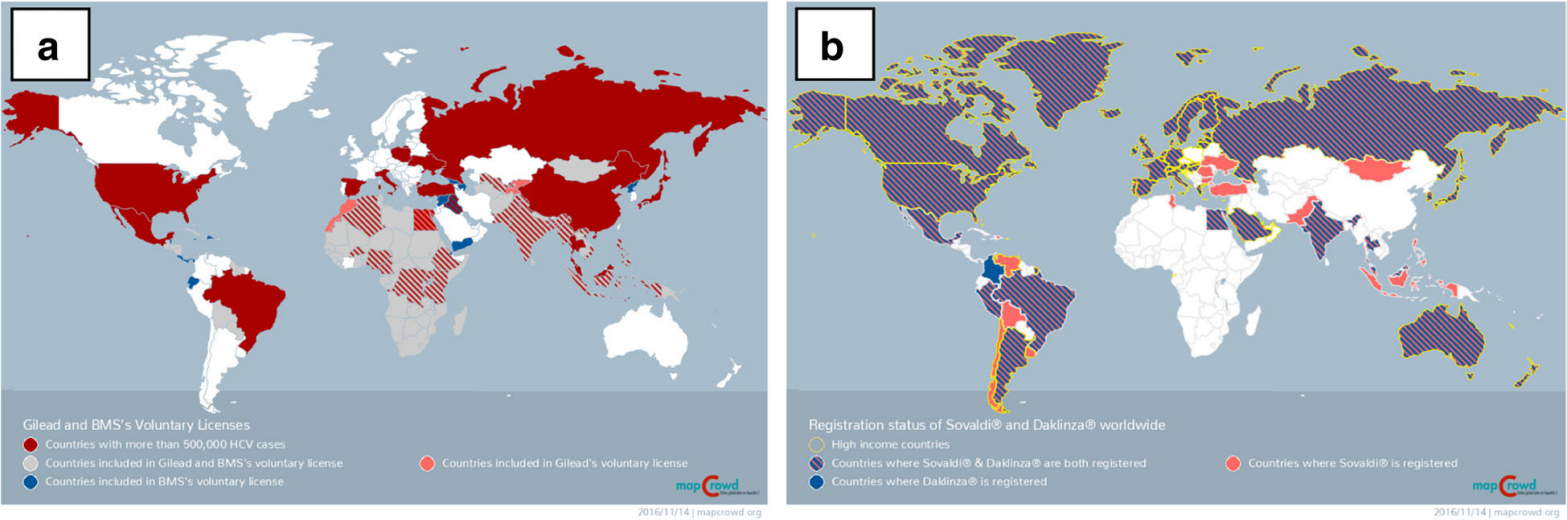
**a** shows countries covered under voluntary licensing agreements from Gilead Sciences and Bristol-Meyers Squib, while 1B shows the registration status of Sovaldi® (Gilead Sciences) and Daklinza® (Bristol-Meyers Squib) worldwide. The voluntary licensing agreements exclude some countries where the burden of hepatitis C is very high, while these drugs remain unregistered in sub-Saharan African countries and parts of Asia. (Source: http://mapcrowd.org)

With the introduction of generic DAAs, a cure is a reality for people living with hepatitis C. However, the price of originator HCV drugs is high and beyond the reach of many patients, including those within resource-rich settings [46]. Drug originators and the Medicines Patent Pool should ensure that their voluntary licenses not only cover as many low- and middle-income countries as possible (currently, these exclude several high-prevalence middle-income countries), but also that the drugs get registered in the countries covered by these licenses (see Fig. 1). When originators do not register their medicines, they should facilitate registration by providing the relevant clinical trials data to licensed generics manufacturers. Quality assurance should also be considered a priority. Currently, only Bristol-Myers Squibb has received WHO prequalification for daclatasvir under the WHO Prequalification of Medicines Programme. This is despite the fact that daclatasvir production is sub-licensed to seven generic manufacturers through the Medicines Patent Pool, and that most of the anti-hepatitis C drugs are on the WHO Model List of Essential Medicines.

### New tools and new technologies

Viral hepatitis-related diagnosis and treatment services do exist, but access to these is a considerable challenge. There is a need for innovative models that could improve diagnosis and treatment in resource-limited settings. Such models include public-private partnerships to unlock funding, and point-of-care diagnostic testing that could be used in remote settings, enabling linkage to care and treatment and also reducing loss to follow up of infected patients. In designing new technologies for diagnosis, it is worth considering the potential of technologies such as loop mediated amplification testing for viral nucleic acid, which can be performed using simple equipment, with results being read using the naked eye [47]. Again, these would be useful in remote settings where there is no specialized equipment similar to that seen in centralized laboratories.

Furthermore, non-invasive tests for screening of liver diseases are required when initiating and monitoring response to therapy and also for monitoring of disease progression in patients with viral hepatitis [48]. There are two general classes of non-invasive tests, namely serum-based tests and radiological tests, as well as a combination of both. The WHO Diagnostic Guidelines, drafted in the context of the WHO Global Health Sector Strategy on Viral Hepatitis 2016–2021 [3, 42], emphasize the fact that testing for viral hepatitis is crucial because it is at the core of the care, treatment and prevention cascade. The WHO Diagnostic Guidelines focus on lower-income countries and adopt a public health approach, promoting standardized, simplified, cost-effective, equitable and feasible approaches to dealing with the burden of undiagnosed and untreated viral hepatitis infection.

## Conclusions - overcoming remaining challenges

More reliable and detailed data on key populations and access to diagnostic platforms, including point-of-care tests and treatment, are needed, as is increased public awareness. Accessible, affordable and caring health services are necessary to strengthen screening, diagnosis, treatment and prevention of viral hepatitis. Civil and community activism can be re-energised as the tools exist, but their effectiveness is hindered by a lack of awareness and political commitment. On this point, lessons could be learned from the HIV response in terms of access to affordable health services, awareness and PMTCT. What is needed now is concrete and concerted action from all stakeholders to eliminate viral hepatitis as a public health threat.

There is a shift to take a public health approach and focus on resource-limited settings to deliver cost-effective, simplified and standardized treatment and prevention national programs and surveillance strategies. The fact that hepatitis C can now be cured should provide encouragement. However, several challenges remain, including the need for more WHO prequalified HCV drugs, the need for originators to ensure access to their drugs by registering (or at least facilitate registration) in all countries, and the need for improving access to treatment for people with HBV mono-infection.

In conclusion, delegates of the 3rd International HIV/Viral Hepatitis Co-Infection Meeting agreed on the following advocacy priorities:

i. Universal implementation of birth dose vaccination for hepatitis B without further delay
ii. Sustainable access to antiviral therapy for HBV mono-infected individuals
iii. Increased diagnosis and treatment of viral hepatitis, particularly HCV among PWID
iv. End of stigmatization of people living with HIV and/or viral hepatitis.

## Abbreviations

DAAs: Direct acting antivirals
HBV: Hepatitis B virus
HCC: Hepatocellular carcinoma
HCV: Hepatitis C virus
MTCT: Mother-to-child transmission
PMTCT: Prevention of mother-to-child transmission
PWID: People who inject drugs
WHO: World Health Organization
